# Going around the bend to understand the role of leg coalescence in metachronal swimming

**DOI:** 10.1242/jeb.249330

**Published:** 2025-06-20

**Authors:** Nils B. Tack, Sara O. Santos, Monica M. Wilhelmus

**Affiliations:** School of Engineering, Brown University, 345 Brook St, Providence, RI 02912, USA

**Keywords:** Pleopod, Differential stiffness, Coalescence, Shrimp, Metachronal propulsion

## Abstract

Many of the most abundant aquatic invertebrates display metachronal swimming by sequentially beating closely spaced flexible appendages. Common biophysical mechanisms like appendage spatial asymmetry and phase drive the success and performance of this locomotor mode, which is generally explained by the need to maximize thrust production. However, the potential role of these mechanisms in drag reduction, another important contributor to overall swimming performance, has yet to be evaluated. We present several morphological, functional and physical mechanisms promoting drag reduction during metachronal swimming by exploring appendage differential bending and leg grouping (coalescence). We performed micro-computed tomography (μCT) and *in vivo* velocimetry measurements of shrimp (*Palaemon vulgaris*) to design a five-legged robotic metachronal analog. This test platform enabled simultaneous flow and force measurements to quantify the thrust and drag forces produced by flexible and stiff pleopods (legs) beating independently or coalescing. We tested the hypothesis that coalescence and bending effectively reduce drag during the recovery stroke (RS). The curved cross-section of the pleopods enables passive asymmetrical bending during the RS to reduce their drag coefficient by up to 75.8% relative to stiff pleopods. Bending promotes physical interactions, facilitating the coalescence of three pleopods at any time during the RS to reduce drag such that the mean net thrust produced during coalescence is increased by 30.2%. These improvements are explained by the production of a weaker wake compared with stiff and non-coalescing pleopods. Our results describe fundamental biological and physical components of metachronal propulsion that may aid the development of novel bio-inspired underwater vehicles.

## Teaser

**Summary:** Shrimp swimming legs bend nearly horizontally and cluster together during metachronal propulsion to reduce drag and improve overall swimming performance.

## INTRODUCTION

Metachronal propulsion consists of sequentially beating closely spaced appendages to generate thrust. Its ubiquity across taxa, including many of the major groups performing diel vertical migrations, such as copepods and krill (Forward, 1988; Mauchline, 1998), suggests common biophysical mechanisms that drive evolution, fitness and ecology. Coordinating several appendages metachronally, rather than synchronously, has been consistently found to promote a higher average momentum flux, maximizing performance during forward swimming (Alben et al., 2010; Byron et al., 2021; Ford and Santhanakrishnan, 2021a; Ford et al., 2019; Herrera-Amaya and Byron, 2023; Khaderi et al., 2012; Peterman and Byron, 2024). Additionally, the induced wake can interact constructively with the downstream appendages, leading to beneficial tip vortex interactions that further enhance thrust (Ford and Santhanakrishnan, 2021b; Garayev and Murphy, 2021). These mechanisms are sensitive to appendage synchronicity (i.e. phase) and spacing, for which most metachronal swimmers fall within a narrow range (Alben et al., 2010; Byron et al., 2021; Ford and Santhanakrishnan, 2021a; Ford et al., 2019; Garayev and Murphy, 2021; Kils, 1981). Metachronal appendages must also achieve significant spatial or temporal asymmetry between the power and recovery strokes (PS and RS) to maximize net thrust (Herrera-Amaya and Byron, 2024; Lou et al., 2022). Existing research has largely demonstrated that metachronal propulsion is a strategy that maximizes thrust production (Alben et al., 2010; Byron et al., 2021; Colin et al., 2020; Ford et al., 2019). But drag reduction is another important factor to consider in self-propelled systems and can be fundamental in promoting net thrust by reducing appendage drag.

At large scales, metachronal crustaceans such as shrimp have complex biramous swimming legs (pleopods) and fine setae that open and close during the power and recovery phases, respectively. Recent studies have highlighted the effect of dynamically modulating the effective surface area (the projected area perpendicular to the flow) of the propulsors by morphing to induce significant spatial asymmetry between the PS and RS (Byron et al., 2021; Lou et al., 2024; Oliveira Santos, 2024; Oliveira Santos et al., 2023; Vogel, 2020). Maximizing thrust and minimizing drag during the PS and RS, respectively, enables metachronal swimmers to effectively overcome both the resisting body drag and the drag on the appendages to achieve propulsion. However, the contribution of other area modulation mechanisms to enhance spatial asymmetry, such as bending during the RS, can play a comparatively critical role.

Metachronal swimmers keep their appendages relatively stiff during the PS but display significant lengthwise bending to minimize the effective surface area, and thus any form drag, during the RS (Herrera-Amaya and Byron, 2024; Herrera-Amaya et al., 2021; Hessler, 1985; Johnson and Tarling, 2008; Lou et al., 2022). Ciliated microorganisms generate asymmetric waveforms by actively sliding adjacent microtubules within the propulsor (Dutcher, 2019). Whether other larger metachronal organisms such as crustaceans achieve active appendage spatial asymmetry using a similar mechanism is unknown. In shrimp, passive asymmetrical bending resulting from the inherent structural or material properties of the appendages is more likely given the high mechanical stability and functional adaptation of crustacean exoskeletal elements through structural and compositional diversity (Fabritius et al., 2016; Raabe et al., 2005). By bending under fluid loading, flexible appendages can reduce their effective surface area and become more streamlined, lowering the drag and stress on the structure (Alben et al., 2002; Gosselin et al., 2010; Vogel, 2020).

Coalescence leverages the interactions between multiple flexible appendages to reduce the total surface area further, lessening interactions with the fluid and potentially lowering drag during the appendage RS. Antiplectic metachronal swimming enables two or more adjacent appendages to coalesce such that their profiles overlap (Byron et al., 2021; Colin et al., 2010; Garayev and Murphy, 2021; Murphy et al., 2011; Sensenig et al., 2010; Tamm, 2014). As posterior appendages enter the recovery phase, they press against the anterior appendages transitioning from the PS to the RS. This is particularly apparent in metachronal animals operating in the transitional to inertial flow regimes with Reynolds number *Re*>10 (Blake and Sleigh, 1974; Byron et al., 2021; Garayev and Murphy, 2021; Murphy et al., 2011). While stiff appendages can still coalesce, by virtue of proximity and phase, interactions within a group might cause destructive collisions or incomplete contact, causing flow instability. Although appendage asymmetrical bending and coalescence can be observed in freely swimming animals, we still need a mechanistic understanding of the relationship between flexibility, temporal coordination and coalescence.

Efforts to quantify processes in how krill generate thrust and lift have successfully measured the momentum of the wake (Ford and Santhanakrishnan, 2021b; Ford et al., 2019; Murphy et al., 2013). Building on these findings, computational fluid dynamics (CFD) models have provided further insight into how metachronal swimmers exploit a vortex-weakening mechanism to produce less overall drag and improve the thrust-to-power ratio significantly (Lionetti et al., 2023; Lou et al., 2024). Grouping multiple flexible appendages was found to facilitate their recovery by allowing edge vortices to merge, causing a significant reduction in pressure in front of the appendage by redirecting the anterior flow in the stroke direction more effectively (Lou et al., 2025). Still, the contributions from individual and interacting legs and the independent effects of appendage bending and coalescence on the swimming performance were not isolated. Recent studies using robotic metachronal systems fitted with segmented appendages (Chen et al., 2023) and magnetoactive soft appendages (Peterman and Byron, 2024) recognized the importance of achieving asymmetry and maintaining a particular inter-appendage phase to induce a sufficiently large net thrust to drag differential between the PS and RS and achieve forward swimming. Work on live ctenophores showed that appendages oscillating with spatial asymmetry can redirect force vectors and enhance propulsive efficiency (Herrera-Amaya and Byron, 2024), further underscoring the importance of asymmetrical bending in metachronal propulsion. At larger scales, how flexible bending of appendages may contribute to drag reduction has thus far been assessed by simplifying appendage morphology as rigid or by using hinged plates (Ford and Santhanakrishnan, 2021a; Murphy et al., 2011; Ruszczyk et al., 2022).

This investigation presents several morphological, functional and physical mechanisms promoting drag reduction during metachronal swimming by establishing the relationship between appendage flexibility, temporal coordination and coalescence. Using micro-computed tomography (μCT) measurements, we examined the morphology of marsh grass shrimp (*Palaemon vulgaris*) pleopods to explore the characteristics enabling passive asymmetrical bending. We conducted free-swimming, *in vivo* kinematics and velocimetry experiments to quantify bending and coalescence and visualize their hydrodynamics. Using these biological data and leveraging our Pleobot (Oliveira Santos et al., 2023), we designed a scaled metachronal robot with five morphologically accurate and flexible pleopods capable of asymmetrical bending. We measured the thrust and drag of independent and coalescing pleopods and evaluated the resulting wake using simultaneous force and fluid flow measurements. We compared these results against rigid pleopods to answer the following three questions. Does pleopod bending significantly reduce drag during leg recovery? Given that shrimp group three recovering pleopods together, does coalescence turn the drag of several appendages to that of only one? How complementary are bending and coalescence in optimizing drag reduction? We provide evidence that reducing drag, rather than simply enhancing thrust, is a determinant factor in the functional morphology and coordination of metachronal propulsors. Our results highlight unifying biophysical principles of this locomotor mode that can be applied to designing novel bioinspired underwater robots.

## MATERIALS AND METHODS

### Animals

Adult marsh grass shrimp (*Palaemon vulgaris* Say 1818) (*n*=13; mean±s.d. body length 3.11±0.25 cm) were collected in June 2022 from Narragansett Bay (Rocky Point State Park, Warwick, RI, USA). The shrimp were housed at room temperature (21°C) in a 38 l aquarium with 30‰ salinity. The capture and experimentations were conducted in accordance with the laws of the State of Rhode Island.

### μCT scan

A shrimp specimen was euthanized in a solution of 25% ethanol (seawater, 30‰) and transferred to a 70% ethanol solution for 20 min until rigor mortis occurred. The left and right pleopods (from P1 to P5) were amputated at the proximal end of the protopod. This was done to separate the pleopods to avoid physical interactions and to enhance the clarity of the resulting high-resolution μCT scan. The samples were preserved in 100% ethanol for 5 days and stained in a 1% solution of iodine in 100% ethanol for 24 h. They were then submerged in 98% hexamethyldisilazane (HMDS) for 24 h and air-dried overnight. Chemical drying using HMDS produces similar results to critical-point drying but allows larger samples to be processed (Alba-Tercedor and Alba-Alejandre, 2019; Bhattacharya et al., 2020). The samples were then mounted onto a custom-made 3D-printed mount that fitted the holding tray of the μCT scanner. A SkyScan 1276 high-resolution microtomography (Bruker, Billerica, MA, USA), upgraded to receive a Hamamatsu microfocus X-ray source (L10321-67, Bridgewater Township, NJ, USA) and a 4K camera (XIMEA MH110XC, Lakewood, CO, USA), was used to perform high-resolution scans (see Supplementary Materials and Methods for specific scanning parameters). We used the Bruker micro-CT Skyscan software (NRecon, DataViewer, CTAnalyser) for primary 3D reconstructions from cross-section images. Volume rendering images were obtained with Slicer v.5.0.3. The 3D reconstruction was used to extract transverse sections of the endopodite and exopodite of the ten legs of the specimen (pooling left and right appendages) to measure the radius of curvature of the chord, *R*, giving the chordwise curvature κ as its reciprocal (see details in the Supplementary Materials and Methods). Curvature was measured from chordwise cross-sections halfway through the length of each ramus (Fig. S1). To account for differences in the chord (distance between the medial and lateral edges) of the ramal structures that proportionally impact the raw value of the radius of curvature, we normalized κ to the chord, *W* of the ramal structures (established from the above transverse sections) as κ=*W*/*R*. Note that we excluded the chordwise curvature of the left and right P1 endopodites from analyses because they are sexually dimorphic, atrophied and not involved in propulsion.

### Pleopod flexural stiffness

Five specimens were euthanized and all their pleopods were amputated at the proximal end of the protopod and were used within 1 h. The fresh protopod of each pleopod was fixed to a rigid horizontal metal support pin using cyanoacrylate glue. The peduncle–ramal joint was immobilized by depositing a minute amount of glue on the anterior face of the joint. This was done to eliminate the effects of passive joint flexing during force measurements. The orientation of the pleopod was established consistently prior to mounting to obtain comparable measurements across all legs and was confirmed visually before each measurement. In all cases, either the endopodite or the exopodite of one leg in a pair was randomly amputated to avoid mechanical interactions during testing. The second ramal structure was isolated similarly using the opposing leg. The mounted pleopods were immersed in 30‰ salt water to maintain the natural material properties and were placed on a precision balance (VWR-124B2, VWR International, precision 0.1 mg). Using a second pin, we applied a series of vertical point force loads at the appendage tip on the ventral and dorsal sides, corresponding to the PS and RS, respectively. Specifically, we applied the point force at the tip of the membranous part of the rami rather than at the tip of the projecting distal setae. This is because the setae have different material properties and spread out, thus letting the pin through. Five replicates were obtained on both sides of each structure, and two pictures were taken for each replicate, starting with the unloaded state and then the loaded state. We analyzed each image of the deflected pleopod unit using MATLAB R2022a (MathWorks, Natick, MA, USA) to determine its effective beam length and deflection. Beam length (*L*, in m) was measured as the distance from the peduncle–ramal joint to the point of applied force. Deflection (δ, in m) was measured as the mean vertical distance traveled by the tip of the appendage from its initial reference location for each set of five replicates. Overall lengthwise flexural stiffness *EI* was measured using the beam equation as:
(1)

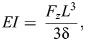
where *F_z_* is the product of the mass reported on the balance readout and the gravitational acceleration (in N). This equation measures the flexural stiffness over the entire beam length and assumes that the beam is homogeneous. Because the equation applies only to small displacements, we removed any measurements for pleopod displacement δ>0.08*L*. This is because if δ is large relative to *L*, the beam is bent down so far that its coordinates in the *x*-dimension while loaded are significantly different from its coordinates while unloaded, thus invalidating measurements (Combes and Daniel, 2003). Eqn 1 is valid for our measurements because even in the most extreme cases (where δ is near 0.08*L*), the error in coordinate introduced by length changes in the *x*-dimension was less than 0.3% (equivalent to the measurement error).

### Biological flow visualization

Biological fluid flow experiments were performed with free-swimming shrimp specimens in a quiescent 9.5 l aquarium measuring 31.1×15.9×20.6 cm (internal length×width×height) with a salinity of 30‰ and temperature of 21°C. Individual shrimp were brought downstream of the field of view (FOV width≈2 body lengths) and within the focal plane of the camera using a beaker. The center of the tank was coincident with the center of the field of view and the focal plane, yielding a clearance from the walls of about 6 body lengths anteriorly, 15 body widths laterally and 10 body heights vertically. As setting up each trial induced disturbances in the flow, recording was performed only when the background flow had subsided to a minimal observable level. Individual shrimp were allowed to exit the beaker and swim freely through the field of view, reaching constant speed by the time they entered it. We first conducted a visual assessment during the experiments to identify steady swimming cases and kept only the sequences of interest, retaining up to ten videos per specimen. We then used a custom MATLAB script to track surface features of the cephalothorax, which allowed us to confirm instances of steady swimming. We selected a single video per specimen for subsequent analyses based on specific criteria, including the clearest example of forward steady swimming and sequences where the left pleopods (closest to the camera) remained within the focal plane throughout the duration of the sequence. We performed 2D bright-field particle image velocimetry (PIV; also known as particle shadow velocimetry) by seeding the water with 10 μm particles (Dantec Dynamics, Skovlunde, Denmark) and back-lighting the animals with an LED illuminator (M530L2-C3, Thor Labs, Newton, NJ, USA) coupled with a collimating Fresnel lens (focal distance 10 cm) to achieve high contrast between the particles and background and uniformly light the domain. We recorded images from the lateral view (corresponding to the left side of the animals) using a high-speed digital video camera (Fastcam Nova R2, Photron, Tokyo, Japan) at 2000 frames s^−1^ at a resolution of 2048×1472 pixels. The camera was fitted with a 60 mm macro lens (Nikon, AF Micro Nikkor, Nikon, Japan) set at f/5.6, yielding a depth of focus of ∼57.1 μm and measurement plane width (i.e. depth of correlation) of 357.3 μm, approximately 60% of the pleopod distal ramal chord (see Supplementary Materials and Methods). Although the shallow correlation depth is sufficient to capture reliable 2D PIV measurements at both the appendage and whole-body levels, it does not entirely eliminate the influence of the 3D flow generated by the substructures of each pleopod. Nevertheless, this potential limitation is mitigated by the known periodicity and symmetry of the flow around the pleopods, which ensures that our approach provides adequate resolution to accurately assess the dominant flow patterns at both the appendage and system-wide scales.

Fluid velocity vectors were calculated from sequential images analyzed using the DaVis 10 software package (LaVision, Göttingen, Germany). Image pairs were analyzed with three passes of overlapping interrogation windows (75%) of decreasing size from 64×64 pixels to 48×48 pixels. All frames were used for the analysis, yielding a separation between frames (d*t*) of 5×10^–4^ s. Velocity fields were input to a custom program in MATLAB 2022a (MathWorks) that computed the corresponding vorticity fields.

### Shrimp kinematics and morphometrics

The shrimp pleopod kinematics data were extracted from image sequences using a custom program in MATLAB. The profiles of individual pleopods (from P1 to P5) of the left side of each shrimp were digitized manually. Because the protopod is rigid, it was digitized as a straight segment, while the endopodite was digitized as a Bezier curve. This method accurately matches the deformation of the pleopods during both the PS and RS. We digitized the endopodite because it remains aligned with the focal plane, unlike the exopodite, which rotates out of plane. Every 10 frames were used for analysis, yielding a separation between frames (d*t*) of 5×10^–3^ s. Pleopod lengthwise bending during swimming was quantified using the curvature along the ramal structures for each leg. Points along the pleopod of length *L* were specified as fractional distance from the peduncle–ramal joint. The radius of curvature *R* was measured at all points along the pleopod Bezier profiles. We used curvature, κ (the reciprocal of *R*), normalized to pleopod length *L*.

The α angle was calculated by measuring the angle between the protopod and a line passing through the proximal joint of the protopods of P1 and P5 (aligned in the direction of swimming). The β angle is traditionally measured between a line passing through the proximal and distal sections of the ramal structures. However, because the endopodite and exopodites bend, we instead measured β between the protopod and a line tangent to the proximal section of the endopodite (corresponding to 20% of the total length of the endopodite) to capture only the movement due to the peduncle–ramal joint. A fast Fourier transform of the α angle over time was used to determine the sequence-averaged pleopod beat frequency. The appendage *Re* – averaged across P1–P5 (*n*=6 shrimp) – was computed from the measured kinematics as *Re*=*U*_tip,max_*L*/ν≈1720, where *U*_tip,max_ is the maximum linear pleopod tip speed derived from the pleopod kinematics and ν is the measured kinematic viscosity of 30‰ seawater at 21°C (ν=1.019×10^–6^ m^2^ s^−1^). We chose this approach to establish the highest *Re* (and thus the highest initial forces) the flexible appendages of the robotic analog would experience. This information was essential for determining the appropriate materials and guiding the fabrication process. The Reynolds number is often defined as *Re*=*VL*/ν=2π(θ/360)*fL*^2^/ν, where the characteristic velocity *V* is the mean pleopod tip speed corresponding to the product of the stroke frequency (*f*) and arc length [2π*L*(θ/360)] and θ is the stroke amplitude of each appendage (in degrees). To allow comparisons with previous studies of other metachronal swimmers using this definition, our appendage *Re* was approximately 310.

Shrimp pleopod coalescence was quantified through the coalescence ratio (CR) for each pleopod, the ratio of pleopod grouping duration during a beat (when in contact with at least one adjacent pleopod) to the duration of the corresponding RS. Coalescence was discretized using three categories: leading (when the pleopod in a coalescing group is facing the flow), confined (when the pleopod is surrounded by an anterior and posterior pleopod) and trailing (when the pleopod in a group is posterior to the others). We defined the metachronal wave period (*T*_wave_) as the time between the beginning of the PS of P5 (initiating the wave) and the end of the RS of P1 (ending the wave) after all the other pleopods have completed their cycle. For P2–P4, with anterior and posterior neighbors, coalescence starts when a recovering posterior pleopod contacts the posterior face, followed by when the anterior pleopod contacts the anterior face upon finishing its PS. The phase φ between two consecutive pleopods was measured as the time between the start of the PS of a corresponding pleopod relative to that of its posterior neighbor (the latter propagating the metachronal wave earlier) and was normalized to its beat period (calculated as 1/*f*). The ratio of pleopod spacing (*B*) to pleopod length (*L*) was calculated as the distance between the proximal protopodite joint of a corresponding pleopod and that of its posterior neighbor, divided by the sum of its protopodite and exopodite lengths. Because P5 does not have a posterior neighbor, *B*_P5_ was measured as the distance between P4 and P5.

### Robotic analog

**Fig. 1.**
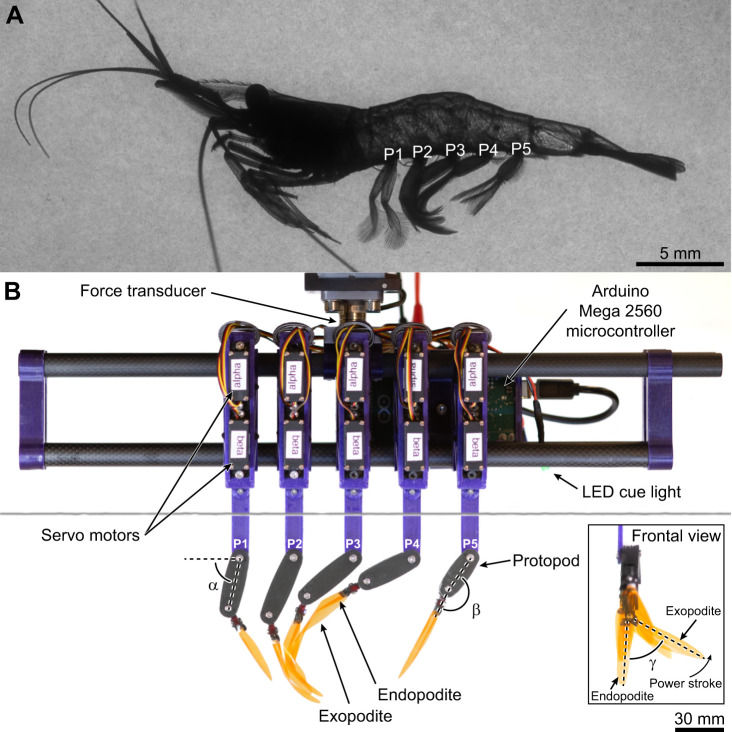
**Biological and robotic models.** (A) Representative free-swimming marsh grass shrimp (*Palaemon vulgaris*). (B) The tethered bioinspired 20× robot with five flexible appendages replicates pleopod bending and coalescence, and measures forces. We used pleopods representative of the left side of the animal, consistent with kinematics data of free-swimming shrimp. Pleopods are identified from P1 (anterior) to P5 (posterior). The α, β and γ angles are shown for reference. The horizontal gray line is the fluid surface.

We used the mean shrimp leg morphometrics (*n*=10 specimens) and kinematics data for the α and β angles of one representative shrimp to design and operate a 20× scaled 3D-printed five-legged shrimp robotic analog (Fig. 1, Table 1). The kinematics data from a single shrimp specimen demonstrating horizontal steady swimming were used to drive the robotic leg motions with no destructive interference between pleopods, as averaging data from multiple specimens masked individual variations and caused physical contact issues (see Supplementary Materials and Methods). The design and actuation of the legs are based on the Pleobot (Oliveira Santos et al., 2023), whose kinematics and hydrodynamics have been validated experimentally against biological data. Here, we fitted the robot with flexible ramal structures made from plastic shims with constant thickness (Practi-Shim™, AccuTrex Products Inc., Canonsburg, PA, USA; thickness 0.0254 mm), whose chord profile was heat-shaped using the same curvature profile of shrimp pleopods to achieve differential stiffness (see Table 1; Supplementary Materials and Methods, Fig. S2). Implementing chordwise curvature effectively replicated the asymmetrical bending observed in shrimp pleopods. Although this approach did not fully capture the complexity of shrimp appendages, it provided a functional model that was stiff during the PS and flexible during the RS, demonstrating that chordwise curvature alone was sufficient to achieve the required bending behavior. Multiple factors beyond geometry (i.e. material properties, density, elasticity and surface pattern) likely influence differential stiffness in shrimp; hence, the flexural stiffness of the robotic pleopods was not characterized as it would not yield a relevant comparison to shrimp pleopods. Our design approach was motivated by the need to achieve, rather than optimize, asymmetrical bending. Note that because the P1 endopodite is sexually dimorphic, atrophied and not involved in propulsion in *P. vulgaris*, the robotic P1 pleopod did not include an endopodite. The viscosity of the fluid and the beat frequency of the legs were adjusted to match the *Re* of the appendages of the five-legged robot to that of the marsh grass shrimp used in *in vivo* experiments (*Re*_robot_≈*Re*_shrimp_=1720, see Supplementary Materials and Methods, Fig. S3). A 3:2 glycerin:water mix with a density of ρ=1160 kg m^−3^, dynamic viscosity μ=0.0114 kg m^−1^ s^−1^ and kinematic viscosity ν=9.8652×10^–6^ m^2^ s^−1^ was used in the experiments. For the fluid dynamic characteristics of the flow to match, the Strouhal number (*St*) must also be matched. We defined *St*=*fl*/*V* where *f* is the dominant frequency in the flow (equivalent to the beat frequency) and *l* and *V* are the characteristic length and velocity, respectively. We used the maximum velocity in the PS wake (*V*_wake_) as the characteristic velocity for swimming speeds (Ford and Santhanakrishnan, 2021a, 2025; Murphy et al., 2013). For the characteristic length, we used the horizontal distance traveled by the pleopod tip during the PS [*l*=2*L*sin(θ/2)]. Using this approach, the *St* of individual legs ranged from 0.3<*St*<0.6 for the flexible pleopods and 0.3<*St*<0.7 for the stiff appendages. While we lack biological data to compare the *St* of independent legs, the *St* of the robotic appendages overlaps with the range 0.2<*St*<2 reported for diverse metachronal swimmers (Garayev and Murphy, 2024).

**Table 1.** Comparative leg morphometrics of *Palaemon vulgaris* and the robotic analog

Model	Parameters	P1	P2	P3	P4	P5
*P. vulgaris*	Mean protopodite length (mm)^a^	2.12±0.11	2.45±0.15	2.36±0.18	2.08±0.11	1.71±0.11
	Mean endopodite length (mm)	N/A	2.26±0.19	2.45±0.25	2.44±0.17	2.06±0.13
	Normalized endopodite chordwise curvature^b^	0.61^c^	1.27	1.31	1.03	1.44
	Mean exopodite length (mm)	2.56±0.26	2.58±0.25	2.89±0.22	2.93±0.22	2.60±0.25
	Normalized exopodite chordwise curvature^b^	1.27	1.24	1.07	1.00	0.99
	Pleopod spacing (mm)^d^	N/A	1.64±0.17	1.8±0.10	1.84±0.13	1.76±0.17
Robot	Scale	20	20	20	20	20
	Protopodite length (mm)	42.4	49.0	47.3	41.5	34.3
	Endopodite length (mm)	N/A	45.1	49.0	48.9	41.1
	Normalized endopodite chordwise curvature^e^	N/A	1.27	1.31	1.03	1.44
	Endopodite chordwise radius (mm)	N/A	7.7	7.7	9.3	5.4
	Endopodite surface area (mm^2^)	N/A	323.54	358.96	341.63	233.85
	Exopodite length (mm)	51.2	51.6	57.8	58.5	52.0
	Normalized exopodite chordwise curvature^e^	1.27	1.24	1.07	1.00	0.99
	Exopodite chordwise radius (mm)	7.9	10.0	13.1	14.1	12.8
	Exopodite surface area (mm^2^)	380.47	473.15	596.99	609.09	487.29
	Leg spacing^d^ (mm)	N/A	32.7	36.1	36.9	35.3

Mean protopodite, endopodite and exopodite lengths for *P. vulgaris* are reported ±s.d. (*n*=10 shrimp). ^a^Measured between the proximal and ramal joints. ^b^Based on the mean of the micro-CT scan of the left and right pleopods of one shrimp. ^c^The P1 endopodite is sexually dimorphic and atrophied in marsh grass shrimp and is not involved in swimming. It was excluded in the robot. ^d^Pleopod spacing reported as the spacing between the particular leg and its anterior neighbor; measured at the center of two adjacent protopodite proximal joints. ^e^The robotic endopodites and exopodites have the same chordwise curvature as that found in marsh grass shrimp.

We performed synchronous high-speed 2D PIV and force measurements to temporally match the kinematics and PIV sequences to the resulting instantaneous forces produced by and acting upon the pleopods. Measurements were acquired after at least ten consecutive metachronal waves to ensure the flow was fully established. Net forces were measured by a 6-axis Nano 17 force transducer (ATI Industrial Automation, Rochester Hills, MI, USA) from which the robot was suspended. The instantaneous force measurements reported in the phased-averaged data depict the net force resulting from the thrust produced by the model and the drag acting on it. The transducer was interfaced with a data acquisition (DAQ) system (NI USB-6210, National Instruments, Fort Worth, TX, USA) outputting data to a custom MATLAB script that converted raw voltages to force data. The sensor bias was measured before each experimental trial and leg conformations, and subtracted from the force data to eliminate the potential effects of sensor drift over time. The robot was initially tested in air to measure the inertial effects during leg motions. However, because of the low beat frequency and the fact that the pleopods have low mass by virtue of being made of thin plastic shims, the forces due to inertia were too small to be distinguishable from the static noise produced by the force transducer under its minimum measurement threshold (uncertainty 2.25 mN). Therefore, we considered inertial effects to be negligible in the experiments.

We used high-speed 2D PIV to compute velocity fields around the robotic legs. Experiments were carried out in a 210 l aquarium measuring 119×43×41 cm (internal length×width×height). The robot was suspended above the surface of the fluid with its pleopods fully submerged. Lateral recordings were acquired by a high-speed, high-resolution digital video camera (Fastcam Nova R3, Photron, Tokyo, Japan) at 500 or 750 frames s^−1^ (4096 pixels×2304 pixels). Seeding particles (10 μm Dantec Dynamics, Skovlunde, Denmark) were illuminated by two adjacent coplanar vertical laser sheets (532 nm, 4000 mW, OptoEngine LLC, Optotronics LLC, Mead, CO, USA) to ensure a uniform particle density field along the entire length of the robot. Both laser sheets pointed upward along the medial plane of the robot (vertical plane passing through the tips of the endopodites).

Several leg configurations were tested to isolate the effects of asymmetrical bending and coalescence on the fluid flows and forces. For instance, we tested individual pleopods (P1–P5) fitted with flexible or stiff appendages to determine the thrust and drag generated in both cases. The force transducer was positioned above the center of mass of the robot and force measurements for individual appendages were obtained using the entire assembly. Because the recorded axial forces are unaffected by the appendage distance from the transducer, the measurements accurately reflect the forces produced by each appendage, even though they are recorded at the whole-body level. Each pleopod was tested independently by keeping all the other legs horizontal, out of the way and influence of the flow produced by the selected pleopod. To investigate the effects of coalescence, we performed five-legged and three-legged experiments. The five-legged cases were analogous to the free-swimming marsh grass shrimp, while the three-legged cases aimed at precisely reproducing a unique coalescing group of three adjacent pleopods. This was done to eliminate the effects of the other legs external to the coalescing group of interest. We report data for the P2–P3–P4 coalescing group. For tests involving multiple beating appendages, the setup could not directly measure forces at the appendage level but rather the net contribution from all the legs at the whole-system level. To test the effect of coalescence on the flow and forces specifically, we compared the results with the cumulative instantaneous forces produced by the same, but independent, legs, time synchronized to the corresponding metachronal wave of the coalescing group. Adding the axial force produced by the individual legs, we effectively approximated appendage-level forces in a multi-legged system with no significant inter-appendage physical or hydrodynamic interactions. This approach allows for comparison with cases where both physical and hydrodynamic interactions take place, such as during coalescence.

The theoretical thrust when coalescence is not occurring was calculated as the cumulative axial force produced by the individual legs. We also tested cases when the metachronal wave was phase-shifted by 180 deg. These unnatural kinematics caused leg contact during the PS but complete separation of the pleopods during the RS. Lastly, to explore the compounded effect of bending and coalescence, we tested the above-leg configurations with flexible and stiff ramal appendages.

Fluid velocity fields were computed using the DaVis 10.2 software package (LaVision, Göttingen, Germany). Image pairs were analyzed with three passes of overlapping interrogation windows (75%) with decreasing size from 64×64 pixels to 48×48 pixels. No smoothing of the velocity data was performed.

The instantaneous force coefficient *C*_F_, whose thrust and drag coefficients (*C*_T_ and *C*_D_, respectively) were of interest in this investigation, was calculated using the axial forces oriented in the swimming direction, corresponding here with the body axis of the five-legged robot. The axial forces generated by individual legs contributed entirely to thrust (in the swimming direction) or drag (opposite to the swimming direction). The signed phase-averaged coefficients were computed over 8–10 consecutive cycles (based on the availability of complete cycles at the beginning and end of the video sequence) from the signed instantaneous force magnitude *F* measured by the load cell, as:
(2)

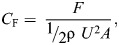
where ρ is the density of the 3:2 glycerin:water mix, *U* is the mean linear appendage tip speed for a stiff appendage calculated using the α stroke amplitude (θ) of the appendage as *U*=2π*Lf*(θ/180) and *A* is the stroke phase-dependent total surface area of the appendage. θ was obtained for each leg from the α angle maxima and minima over several consecutive beats (accounting for the <1 deg orientation bias of the rostro-caudal axis relative to horizontal) using the DLTdv8 package for MATLAB (Hedrick, 2008). This was done by tracking landmarks on the protopodite, such as the distal joint screw. The abduction angle of the exopodite (γ) was not directly measured over time; instead, it was inferred to be at its maximum during the PS and at its minimum during the RS. Consequently, when calculating the phase-averaged *C*_F_, we assumed that the surface area (*A*) was maximal during the PS (γ=60 deg) as the exopodite fully extended (see Fig. 1) and was equal to the combined surface areas of the endopodite and exopodite. In contrast, during the RS when the exopodite adducts and overlaps completely with the smaller endopodite, the total surface area of the pleopod is reduced to the surface of the exopodite only. The coefficients were calculated using the total surface area of the pleopods rather than their effective surface area to the flow – as though both pleopod types were stiff. This is to highlight the expected difference in the drag the stiff and bending pleopods produce (due to different effective surface areas) despite having the same kinematics, shape, size and total surface area.

Thrust and drag were evaluated separately for the thrust-dominated PS and the drag-dominated RS to compare the performance of the flexible and stiff appendages. During the PS, net thrust was always produced, and the mean *C*_T_ was the average positive force during this phase (equal to the mean *C*_F_). To accurately assess drag during the recovery phase, we calculated the mean *C*_D_ by averaging only the negative force values for both flexible and stiff appendages. As the model was tethered, net thrust was artificially produced during the deceleration phase of the RS due to fluid stagnating behind the appendage and producing a forward-directed force resulting in net thrust (Oliveira Santos et al., 2023). This effect, if included when evaluating the *C*_F_ of the appendages, would result in incorrectly interpreting the drag generated during the RS.

### Statistics

All statistical tests were performed using MATLAB 2023b. For biological measurements of the pleopod chordwise curvature, the rami were pooled by type for the only specimen available (for a total of 8 endopodites and 10 exopodites). Note that the sexually dimorphic and atrophied P1 endopodite was excluded from this analysis because it differs substantially from the other legs and is not involved in swimming. Because of the low sample count and as the sample distributions were not normally distributed (one-sample Kolmogorov–Smirnov test, *P*<0.001), we compared the differences between the endopodite and exopodite using a Mann–Whitney *U*-test. Similarly, the flexural stiffness of the anterior and posterior faces of the endopodite and exopodite (*n*=5 shrimp) was compared using a *U*-test for each leg. The relationship between pleopod bending and the angular velocity ω was examined separately for the PS and RS. For the RS, we found that the relationship between the local curvature κ/*L* along the ramus of length *L* pleopod angular velocity is best described by a power fit (κ/*L*=*a*ω*b*+*c*), while the PS is best modeled as a linear fit (κ/*L*=*a*ω*b*+*c*). For robotics experiments, the mean *C*_T_ and *C*_D_ for each consecutive beat cycle (for individual legs fitted with flexible or stiff ramal appendages) were computed for the PS and RS, respectively, and averaged across 8–10 consecutive cycles. We performed *t*-tests to compare the mean *C*_T_ and *C*_D_ of individual flexible pleopods with their stiff counterparts (P1 to P5). Differences were considered significant at *P*<0.05. All the kinematics parameters and force data are reported as means±s.d.

## RESULTS

### Pleopod morphology and asymmetrical bending

**Fig. 2.**
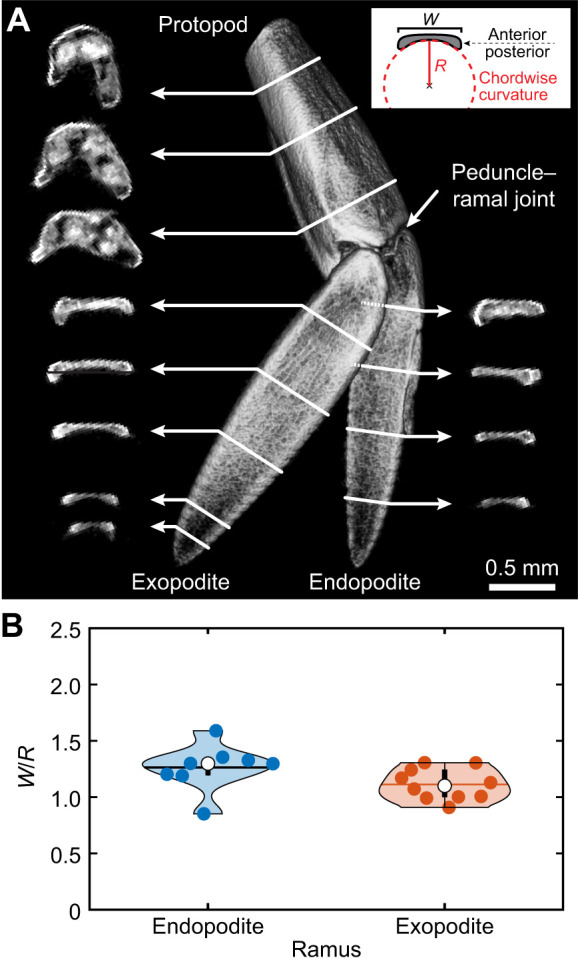
***Palaemon vulgaris* pleopod chordwise curvature.** (A) Reconstructed micro-computed tomography (μCT) scan of the right P2 pleopod of a representative *P. vulgaris* specimen (anterior view). Cross-sections show the chordwise curvature, *W*/*R*, of the posterior face of the exopodite and endopodite, where *W* is the ramus chord and *R* is the radius of curvature (see inset). Note the flat anterior face. The fine setae lining the edges of the endopodite and exopodite were not captured by the CT-scanning process and are thus not visible here. (B) Corresponding mean normalized chordwise curvature of the posterior face of the endopodites and exopodites (*n*=10 legs). Endopodites and exopodites have comparable chordwise curvature (*U*-test, *P*=0.083). The white circle indicates the mean and the horizontal line is the median.

Cross-sections of all the pleopods of one representative *P. vulgaris* obtained from reconstructed μCT scans showed that the posterior face of the ramal structures – endopodite and exopodite – is concave, with pronounced chordwise curvature (Fig. 2A). Pooling the data by ramus type revealed no statistical difference in the normalized curvature *W*/*R* of the endopodites and exopodites (8 endopodites and 10 exopodites, *U*-test, *P*=0.083; Fig. 2B) with *W*/*R*_endopodite_=1.26±0.21 and *W*/*R*_exopodite_=1.11±0.14 (see Table 1 for *W*/*R* of individual pleopods). Note that the chordwise curvature of the left and right P1 endopodites was excluded from analysis because they are sexually dimorphic, atrophied and not involved in propulsion. We observed that the anterior face of the endopodites and exopodites was relatively planar and determined that chordwise curvature was the feature enabling asymmetrical bending. Hence, we simplified the robotic model by only considering the curved profile in the design of the appendages. The rami of each robotic pleopod were shaped using the normalized curvature of the rami of the respective leg measured in *P. vulgaris* (Table 1).

**Fig. 3.**
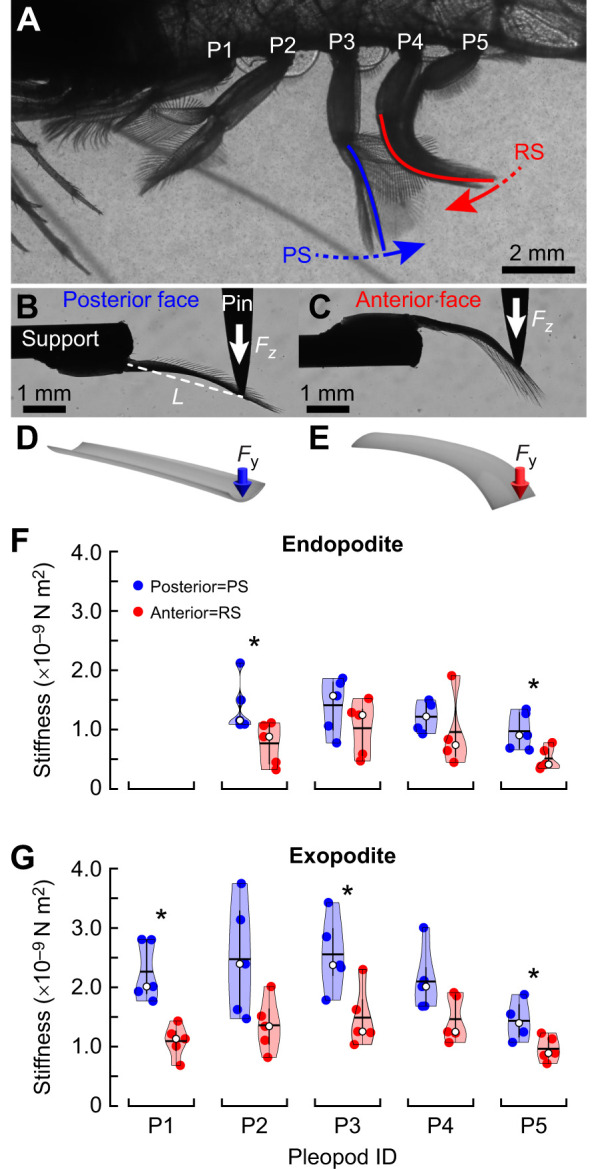
**Differential flexural stiffness of *P. vulgaris* pleopods.** (A) Differential stiffness keeps the pleopods stiff during the power stroke (PS) and allows bending during the recovery stroke (RS). (B) Posterior and (C) anterior measurements of pleopod flexural stiffness. The effective beam length (*L*) is the distance from the peduncle–ramal joint to the point of applied force (*F_z_*). Pleopod deflection is emphasized to illustrate differential stiffness and bending. (D) Conceptually, the concave posterior face stiffens the rami when a force is applied posteriorly (PS) while an anterior force causes bending (E). The endopodites (F) and exopodites (G) were, on average, 1.7 times more flexible during the RS versus the PS (*U*-tests, 0.008<*P*<0.343; *n*=5 shrimp). Asterisks indicate significant differences *P*<0.05 between the anterior and posterior faces. The white circle indicates the mean and the horizontal black line is the median.

The posterior chordwise curvature of the rami enabled the legs to remain stiff during the PS and bend almost horizontally during the RS (Fig. 3A). Flexural stiffness measurements of freshly dissected *P. vulgaris* pleopods showed the rami were, on average, 1.7 times stiffer when a point force was applied posteriorly (corresponding to the PS) compared with the anterior face (analogous to the RS), resulting in pronounced spatial asymmetry (*U*-tests, 0.008<*P*<0.343; *n*=5 shrimp; Fig. 3; Table S1). Stiffness measurements were obtained for the membranous part of the rami and excluded the setae.

**Fig. 4.**
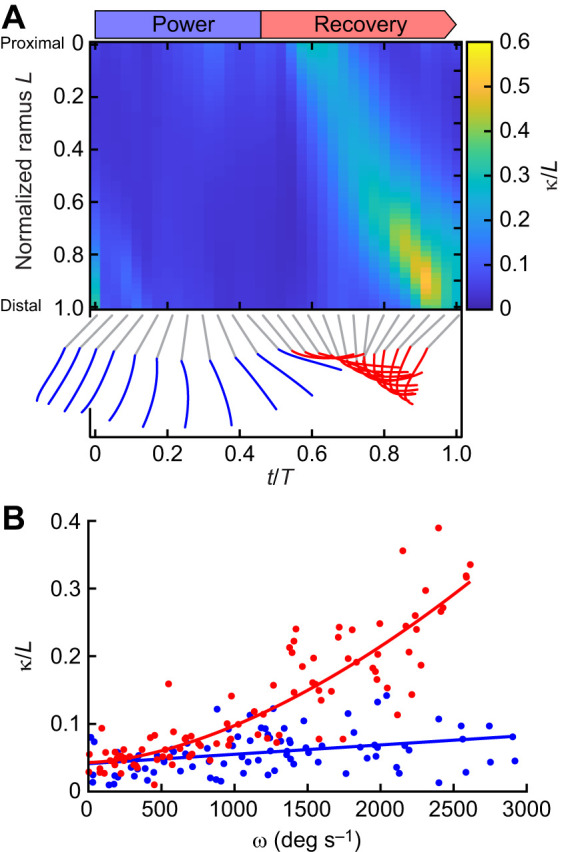
**Passive pleopod asymmetrical bending during a beat for free-swimming *P. vulgaris*.** (A) Local normalized curvature along the flexible P3 exopodite during a full beat cycle. Instantaneous pleopod profiles (blue and red) highlight differences in curvature between the PS and RS. The instantaneous time *t* during the beat cycle is normalized to the beat period *T*. (B) Local lengthwise curvature at 0.3*L* of P3 as a function of angular velocity during a full beat (*n*=6 shrimp). The local curvature increased with the angular velocity (ω) of the pleopod during the RS following a power relationship (κ/*L*=5.84×10^–7^ω1.66+0.04, *R*^2^=0.78). The PS induced little bending and was best modeled using a linear fit (κ/*L*=1.39×10^–5^ω+0.04, *R*^2^=0.122).

Pronounced pleopod lengthwise bending occurred exclusively during the RS, as seen with elevated local curvature propagating from the proximal to the distal sections of the rami of free-swimming shrimp (Fig. 4A; Fig. S4). The rami did not bend along a singular point. Instead, the inflection point induced in the proximal section at the beginning of the RS traveled distally over time until the end of the recovery phase. The rami remained nearly horizontal during the first half of the recovery phase (during the acceleration phase of the recovering pleopod) but eventually unfurled completely in preparation for the following PS (Fig. S5). We found that in *P. vulgaris*, the rami bent passively – rather than actively – as a result of the resistive forces of the flow acting on the anterior face during the RS. We can infer the resistive effects of the flow on the pleopod via the instantaneous angular velocity, as in this regime (*Re*≈1720) drag scales with the square of the velocity (the linear velocity at any point along the length of the rami) of the appendage. By measuring the instantaneous local curvature at a fixed point along the rami during a beat (we selected 0.3*L*, *n*=6 shrimp), we found that lengthwise bending of the rami was proportional to the angular velocity of the pleopod. It was particularly significant during the RS when the local curvature scaled as a power relationship with pleopod angular velocity (κ/*L*=*a*ω*b*+*c*; Fig. 4B; Fig. S4). In contrast, the rami bent only very little with increasing pleopod angular velocity during the PS, which followed a linear trend in this case (κ/*L*=*a*ω+*b*; Fig. 4B; Fig. S4). This is because the pleopod must remain stiff to generate maximum thrust.

**Fig. 5.**
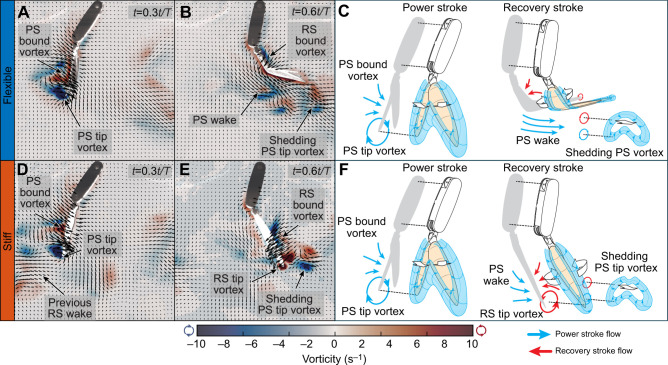
**Instantaneous fluid flow of the flexible and stiff ramal structures in the robotic analog.** (A,B) Instantaneous velocity vectors and vorticity contour fields of P3 fitted with flexible ramal appendages. (C) Simplified schematics of the expected 3D flow around the flexible pleopod. (D,E) Instantaneous velocity vectors and vorticity contour fields of the stiff pleopod. (F) Expected 3D flow around the stiff pleopod. The two appendage types generated comparable flow during the PS (A,D). At the end of the RS, the flexible rami induced a downward, posterior wake (B) while the stiff rami shed backward and forward wakes (E). Every three vectors were plotted for clarity.

**Fig. 6.**
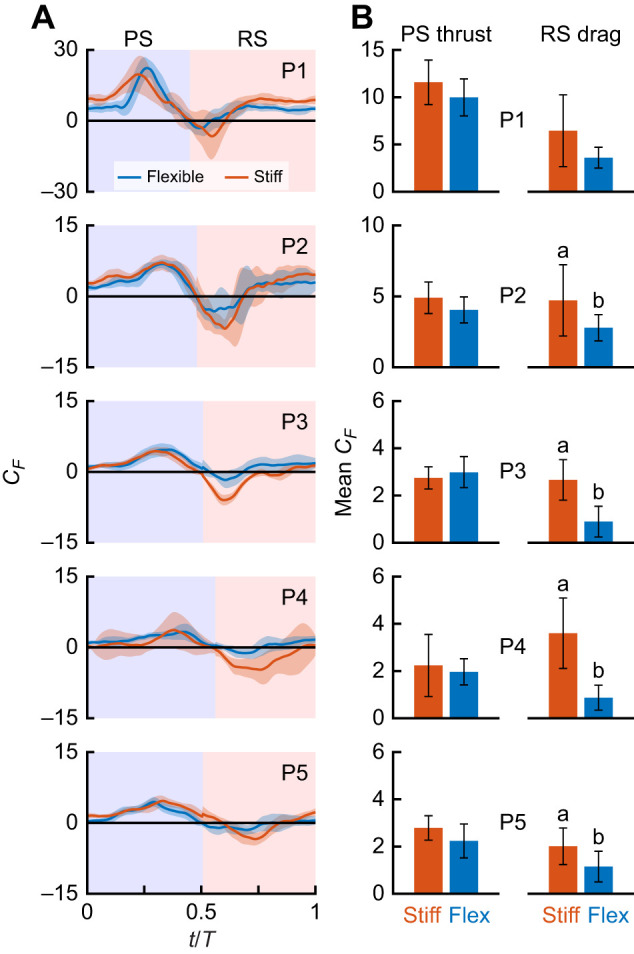
**Flexible ramal structure generates less drag than stiff counterparts in the robotic analog.** (A) Net instantaneous phase-averaged force coefficient *C*_F_ for flexible and stiff P1–P5 (number of consecutive beat cycles, *n*_flex_≤9 and *n*_stiff_≤10). Instantaneous forces depict the net force resulting from the thrust (positive) produced by the model and the drag (negative) acting on it. The colored background indicates the PS (blue) and RS (red). Shading indicates the standard deviation. (B) Comparison of the absolute *C*_F_ during the PS (thrust dominated) and RS (drag dominated). Note that for the PS, net thrust was always produced and the mean thrust coefficient (*C*_T_) was the average positive force during this phase. In contrast, during the RS, the mean drag coefficient (*C*_D_) was calculated as the average of the negative forces throughout the RS. Letters indicate significant differences between force types within a species (*t*-tests, *P*<0.05).

Individual, isolated robotic pleopods fitted with flexible rami behaved similarly to shrimp pleopods and had comparable fluid­–structure interactions to those of their rigid counterparts during the PS (Fig. 5A,D). In both cases, the robotic pleopods remained stiff to sustain the largest surface area and generate maximum thrust. For each pleopod, flexible and stiff rami generated identical PS tip vortices and had equivalent mean PS thrust coefficients (*t*-tests, 0.09<*P*<0.58; Fig. 6). Both cases shed the PS tip vortex from the pleopod tip (Fig. 5B,E). The most notable differences between the flexible and stiff cases emerged during the RS as the flexible rami allowed the PS wake to circulate posteriorly unimpeded (Fig. 5B). In contrast, the stiff rami prevented most of the PS wake from circulating rearward, causing a significant increase in the drag experienced by the leg. By virtue of bending nearly horizontally, the flexible appendages interacted only marginally with the PS wake and had a reduced effective surface area to the flow compared with the stiff rami. This resulted in a significant reduction of the drag coefficient *C*_D_ of P1 to P5 by 30.2%, 41.0%, 66.4%, 75.8% and 42.6%, respectively (*t*-tests, *P*<0.05 for P2–P5 and *P*=0.08 for P1; Fig. 5B). While the flexible rami induced a dominantly rearward wake, the stiff rami shed a RS tip vortex carrying forward momentum (Fig. 5D). We did not find evidence that these flow-induced fluid­–structure interactions impacted either the anterior flow along the pleopod or the thrust coefficient significantly. Overall, both the flexible and stiff pleopods had a PS thrust to RS drag ratio *T*/*D*>1. However, the ratio for the flexible rami was always greater than that of their stiff counterpart. For the flexible and stiff rami, respectively: *T*_P1_/*D*_P1_=3.1±1.4 and 2.7±2.1, *T*_P2_/*D*_P2_=2.7±1.0 and 2.0±0.7, *T*_P3_/*D*_P3_=4.3±2.9 and 1.7±0.3, *T*_P4_/*D*_P4_=4.2±3.0 and 1.1±1.0, and *T*_P5_/*D*_P5_=2.9±1.1 and 2.3±0.8. Note that all the pleopods have a positive *C*_F_ during the second half of the RS, which corresponds to the deceleration phase of the stroke. Notably, P1 and P2 transition from the PS to the RS with a significant positive *C*_F_ compared with the posterior pleopods. This can be explained by the interplay of two opposing flows produced by and acting upon the appendages. First, the anterior flow entrained backward during the PS acts on the anterior side of the appendage, generating a backward-directed drag force. Concurrently, because the system was tethered, the flow behind the appendage at the end of the RS stagnates and pushes against the pleopod, thus exerting a forward-directed thrust force as the appendage transitions from RS to PS. The distinction between P1 and P2 and the other pleopods can be attributed to differences in their kinematics. These anterior pleopods exhibit smaller overall stroke amplitudes and shallower α angles (α<90 deg), which result in a PS wake with a more downward rather than axial orientation. Consequently, the posterior stagnating flow potentially has a relatively greater influence than the anterior PS entrained flow, likely producing proportionally larger thrust for P1 and P2 compared with the posterior pleopods. This is supported in part by the fact that the initial *C*_F_ at the beginning of the PS converges to 0 from the anterior to the posterior pleopods as their respective wake and induced flow show a more rearward orientation along the anterior to posterior axis.

### Pleopod coalescence

**Fig. 7.**
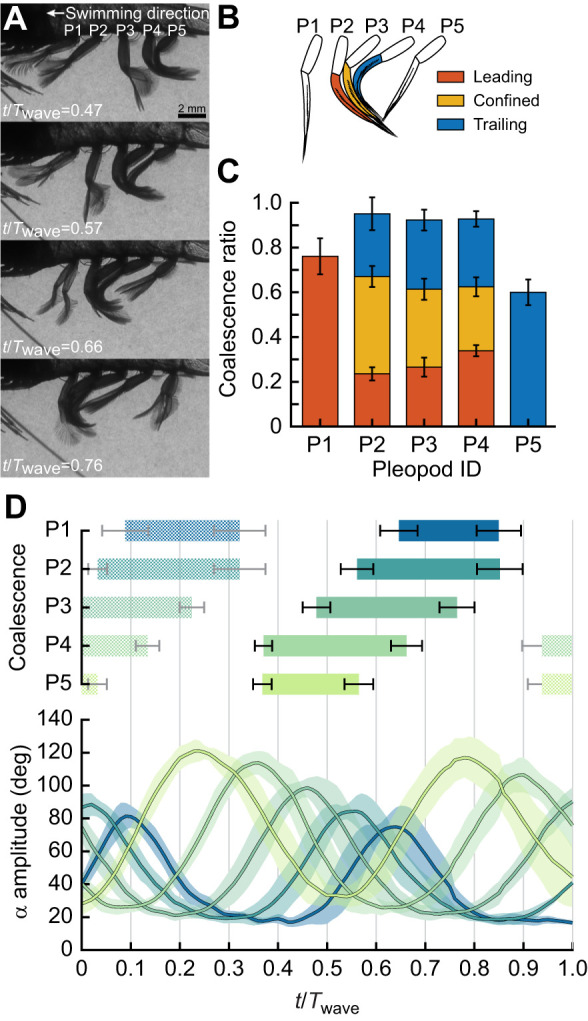
**Pleopod coalescence in free-swimming *P. vulgaris*.** (A) Up to three adjacent pleopods coalesce during their RS. (B) In a coalescing group, the anterior leg leads the movement while the posterior pleopod trails. The pleopod within a coalescing group is confined between the surrounding anterior and posterior legs. (C) The coalescence ratio defines the proportion of the RS during which pleopods coalesce. P2–P4 switched from leading to trailing the group (*n*=6 shrimp). (D) Pleopod coalescence and kinematics visualized through the α amplitude during a full metachronal wave (*n*=6 shrimp). The color code of the pleopod coalescence bars was applied to the α amplitude curves (from light to dark shades from P5 to P1, respectively). Error bars and shading indicate the standard deviation for coalescence and α kinematics, respectively. Textured coalescence bars indicate coalescence occurring during the previous and following metachronal waves.

While pleopod bending plays a crucial role in decreasing the overall drag of each pleopod during the RS, it also enhances leg coalescence. Time series of coalescing pleopods in free-swimming *P. vulgaris* showed that flexible pleopods can wrap around one another and conform to the shape of the surrounding pleopods to enhance coalescence (Fig. 7A; Fig. S5). Another benefit of flexibility is the significant reduction in potentially destructive physical interactions upon collision with one another. Because of the length and proximity of the legs and the phase relative to one another, up to three pleopods form a tight coalescing group. Three-legged coalescence emerges from the specific pleopod separation to length ratio, *B*/*L*=0.364±0.014 (*n*=6 shrimp, all pleopods pooled) and inter-pleopod phase lag φ=0.18±0.02*t*/*T* which causes several legs to perform their RSs simultaneously (Fig. 7D).

**Fig. 8.**
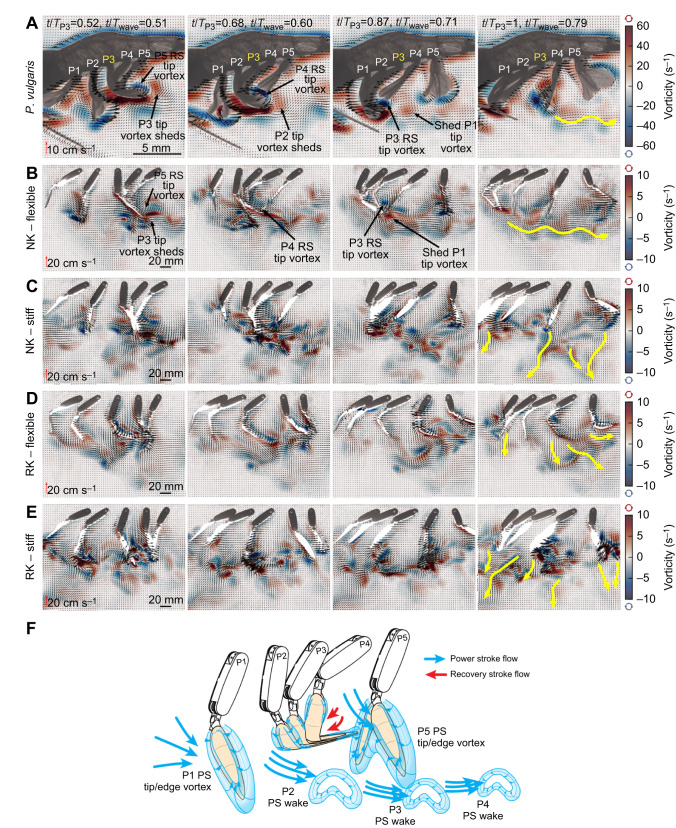
**Velocity and vorticity field time series of *P. vulgaris* and the five-legged robot.** (A) Free-swimming *P. vulgaris* displayed pleopod coalescence during the sequential RS of each leg. Note each pleopod shifting from leading to trailing the coalescing group as the metachronal wave propagates. *T*_wave_­, metachronal wave period. (B) The five-legged robot with flexible rami and displaying natural kinematics (NK) generated a wake analogous to that of *P. vulgaris*. (C) Stiff rami produced a turbulent wake when the recovering pleopods intercepted the PS wake and generated individual wakes with forward and downward components. (D) Flexible rami performing reversed kinematics (RK) without coalescence also had a strong downward component. (E) Flexible RK rami produced wakes with the most forward-oriented component out of all the above cases. Yellow arrows represent the overall direction of the pleopod wakes. For clarity, every two and three vectors are plotted for *P. vulgaris* and the robot, respectively. (F) Simplified schematics of the expected 3D flow around the flexible pleopods displaying coalescence under natural kinematics.

As seen with live animals, the role of each leg changes during a coalescence cycle. For example, as P3 initiates its coalescence cycle, it leads the P3–P4–P5 group and faces the flow directly. Upon reaching the anterior P2, P3 becomes confined between P2 (anterior) and P4 (posterior), both shielding P3 against anterior and posterior flows. The coalescence group becomes P2–P3–P4. Finally, as P4 separates from the P2–P3–P4 group, P3 trails the P1–P2–P3 group, thus becoming exposed to posterior flow. P1 and P5 are exceptions as they only have one posterior and one anterior neighbor, respectively (Fig. 8).

We used the coalescence ratio (CR) to quantify the duration of leg coalescence for a given pleopod relative to the duration of its RS. P2, P3 and P4, which have one adjacent anterior and posterior pleopod during coalescence, have a CR>0.9, with CR_P2_=0.95±0.05, CR_P3_=0.92±0.04 and CR_P4_=0.93±0.03 (*n*=6 shrimp; Fig. 7C). The majority of the RS was dominated by pleopod coalescence. More importantly, each pleopod was confined between two adjacent legs for 43.6±4.7%, 34.8±4.8% and 28.6±4.2% of their RS, respectively, thus guarding individual legs from significant interactions with the surrounding fluid for about 1/3 of the RS. P1 and P5, which led and trailed coalescing groups, respectively, as a result of their anterior and posterior locations, still contributed to coalescence for about 2/3 of their RS, given CR_P1_=0.76±0.08 and CR_P5_=0.60±0.06. *Palaemon vulgaris* displayed more than one metachronal wave at a given time; therefore, several coalescence cycles occurred within the span of a complete metachronal wave propagating from P5 to P1 (Fig. 7D). This further enhanced the overall influence of pleopod coalescence during swimming.

**Fig. 9.**
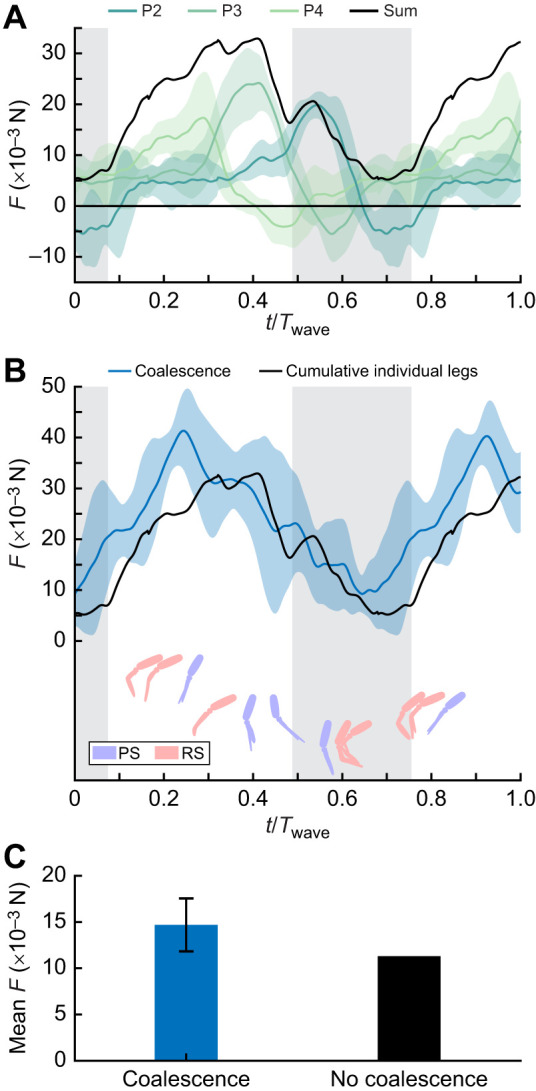
**Comparing the net thrust produced by the robotic flexible coalescing pleopod group P4**–P**3**–P**2 and the cumulative forces from the same but individual pleopods.** (A) Instantaneous and cumulative (black) forces (*F*) produced by independent legs, time synchronized to the corresponding metachronal wave. Colored shading indicates the standard deviation for at least 8 consecutive beat cycles. Background gray shading indicates the coalescence phase. (B) Instantaneous thrust produced by the P4–P3–P2 coalescing group. Coalescence promotes greater net thrust during the coalescing phase (gray shaded background). (C) Coalescing pleopods generate 30.2% more thrust than the same but independent actuating pleopods during the coalescence phase.

Our robotics data emphasized how the inter-pleopod phase and coalescence promote the most consistent rearward flux and fluid momentum to maximize thrust (Figs 8 and 9; Fig. S6). When this arrangement broke down, such as when artificially reversing the metachronal wave to induce pleopod divergence, the wake became more turbulent, the thrust less consistent, and individual pleopod wakes were discernible (Fig. 8; Fig. S6). To investigate how coalescence influences force production, particularly during the phase when the pleopods are closely grouped during their RS, we tested a three-legged configuration (P2–P3–P4; Fig. 9) that replicated the conditions within a coalescing group. We compared the instantaneous forces produced during the coalescence phase by the same, but independent, legs, time synchronized to the corresponding metachronal wave of the three-legged case. We found that three coalescing pleopods generate 30.2% more net thrust than independent pleopods during the corresponding coalescence phase (Fig. 9C).

## DISCUSSION

### The role of pleopod passive bending

Appendage bending is a ubiquitous functional feature of the RS in many metachronal swimmers such as copepods, ctenophores, krill and polychaete worms. This contrasts with the PS, during which the appendages remain relatively stiff to increase the surface area and maximize thrust. The spatial asymmetry of *P. vulgaris* pleopods is primarily driven by the differential flexural stiffness along the posterior and anterior directions (Fig. 3). The rami are, on average, 1.7 times more flexible in the posterior direction – corresponding to the RS when the resistive force of the flow acts upon the anterior face. Using detailed μCT scans of *P. vulgaris* pleopods, we found that the posterior face was concave, with normalized chordwise curvature κ=1.26±0.21 and 1.11±0.40 for the endopodites and exopodites, respectively (Fig. 2). In plants, such chordwise geometry enables different compliances under bending with opposite orientations, with the structure bending toward the concave face (Guzmán et al., 2016; Wei et al., 2022), analogous to the RS in *P. vulgaris*. The curved geometry of the pleopod thus emerges as a passive strategy to effectively achieve spatial asymmetry. The pleopods can bend during the RS when the hydrodynamic forces resisting the motion overcome the inherently weak stiffness given by its concave shape. In contrast, this geometry stiffens the pleopods during the PS when resistive forces act in the opposite direction. To date, there is no morphological evidence suggesting the presence of structures (i.e. muscles, microtubules) capable of inducing controlled lengthwise bending of the pleopod rami via active processes. However, it has been shown that larger organisms can tune their structural (i.e. geometry) and material properties to the inertial forces they experience to achieve passive deformation under strain (Koehl, 1984; Vogel, 2020; Wainwright and Koehl, 1976). Doing so eliminates the need for active control, thus greatly simplifying actuation. The effects of passive bending are further evidenced by the proportional change in local lengthwise curvature with pleopod angular velocity during the RS (Fig. 4). By passively bending under fluid loading, flexible pleopods can autonomously reduce their projected surface area to the flow and become more streamlined during the RS (Vogel, 2020). Self-reconfiguration reduces the stress on the pleopod proportionally to the resistive fluid forces (Alben et al., 2002; Gosselin et al., 2010).

We found that bending lowers the drag coefficient of individual pleopods by 41.0–75.8% depending on the pleopod, compared with their stiff counterpart (Fig. 6). From our robotic experiments, we identified the source of this substantial drop in the induced drag is twofold: (1) bending reduces the effective surface area to the flow and (2) bending mitigates fluid–structure interactions, notably with the rearward-traveling PS wake. Presenting a smaller profile to the flow lowers drag-inducing inertial effects and the nearly horizontal orientation of the pleopod rami further trades inertial effects for much weaker viscous (i.e. shear) forces (Gosselin et al., 2010). Drag reduction is enhanced by limiting interactions with the PS wake that circulates nearly unimpeded posteriorly during the RS (Fig. 5A–C). For comparison, stiff pleopods systematically disrupt the PS wake and induce substantially higher drag (Figs 5E and 6).

Differential flexibility does not significantly affect performance during the PS, as we found no statistical difference in thrust production (i.e. they have similar *C*_T_) between the flexible and stiff configurations. However, the mean *C*_T_ of the stiff appendages appeared slightly higher than that of the flexible legs. This is likely because the stiff appendages present a larger effective surface area to the flow during the RS, resulting in a greater amount of fluid stagnating behind them during the PS to RS transition (marking the start of a new cycle). This increased fluid stagnation can induce slightly higher thrust at the beginning of the cycle, as indicated by the non-zero thrust coefficient at *t*/*T*=0 (see Fig. 6). Additionally, the unbending motion of the flexible appendages during the RS may dampen the forward-directed forces generated by this fluid flow. Consequently, there can be small differences in the PS *C*_T_ between the flexible and stiff appendages, even though both types exhibit similar effective surface areas during the PS. Because of the tethered nature of the model, additional thrust was likely generated during the recovery to PS transition as stagnant fluid behind the appendage produced a forward-directed force. Passive bending helps maximize the thrust-to-drag ratio via spatial asymmetry between the power and recovery phases, enabling shrimp to effectively overcome the resisting appendage and body drag to achieve propulsion (Byron et al., 2021; Herrera-Amaya and Byron, 2023; Lou et al., 2022; Vogel, 2020). Producing less overall drag during the recovery phase reduces the power needed to actuate the pleopod, thus promoting economical swimming and potentially lowering the cost of transport (COT) (Lionetti et al., 2023).

### The role of pleopod coalescence

The phase-dependent grouping of several closely spaced adjacent appendages – coalescence – is intrinsic to antiplectic metachronal waves (Alben et al., 2010; Byron et al., 2021; Ford and Santhanakrishnan, 2021a). The mean inter-pleopod phase φ=0.18±0.02*t*/*T* and pleopod *B*/*L*=0.36±0.01 of *P. vulgaris* promotes the tight grouping of three leg pairs during their recovery phase and the complete separation of the legs during their PS (Figs 7 and 8). This is consistent with the narrow 0.2<*B*/*L*<0.65 and 0.20<φ<0.25*t*/*T* ranges observed in most metachronal organisms (Byron et al., 2021; Garayev and Murphy, 2021, 2024; Murphy et al., 2011). These commonalities are often explained in the context of the PS, for which there is a need to coordinate the swimming appendages and circulate the wake to promote efficient force production (Ford and Santhanakrishnan, 2021a, b; Ford et al., 2019; Murphy et al., 2013). Our data support this idea that the inter-pleopod phase and configuration modulate rearward average flux and fluid momentum to maximize thrust (Fig. 8; Fig. S6).

By promoting inter-pleopod interactions, coalescence can have a significant impact on the performance of metachronal propulsion. For instance, if we reverse the metachronal wave to induce pleopod divergence during the RS, the wake becomes more turbulent and the thrust less consistent (Fig. 8; Fig. S6). Numerical work with ctenophores found that appendage tip vortex interactions between recovering adjacent appendages are necessary to reduce drag and can improve the thrust-to-power ratio by up to 55% (Lionetti et al., 2023). This potential tradeoff between pleopod separation and grouping can explain why metachronal appendages beat within such narrow phase and *B*/*L* ranges.

Coalescence is a pervasive feature of metachronal propulsion, occurring in over 90% of the RS (Fig. 7). While all the legs display coalescence, their position and role within a coalescing group change sequentially from leading, to being confined, to trailing the group as the metachronal wave propagates. The pleopods are thus constantly exposed to changing flow conditions during their beat. Pleopods P2–P4 (which have anterior and posterior neighbors) are completely confined within a group and not directly subject to the flow for about one-third of their RS (Figs 7A,B and 8). To investigate how this affects force production, we tested a three-legged configuration (P2–P3–P4; Fig. 9) that replicated the conditions within a natural coalescing group, thus strictly isolating the effects due to coalescence. We found that net thrust was generated throughout the entire metachronal wave. We compared these results with the time-synchronized force data from the same three independently tested pleopods and found that even without coalescence, only net thrust is produced during a metachronal wave (Fig. 9).

The inter-pleopod phase and spatial asymmetry are sufficient to ensure net thrust is always produced (Ford and Santhanakrishnan, 2021a). Nonetheless, coalescing pleopods produce 30.2% more mean thrust during the coalescence phase than their independent counterparts. Given that the main difference between these two cases is the strong inter-pleopod interactions only during the recovery phase, coalescence emerges as an effective strategy to mitigate drag and improve overall performance. This is attributable to the tight contact between the pleopods, forming a tight seal that shields the coalescing group from flow instabilities that would otherwise develop between the legs (Fig. 8; Fig. S5). We observed that the RS of P5 (initiating the metachronal wave) does not shed a wake in the free stream when coalescence occurs. Instead, the posterior bound vortex is consistently circulated behind the next anterior pleopod as the coalescence group propagates to P1. This means that the traveling bound vortex is trapped between the pleopods and the much stronger inferior, rearward-traveling PS wake, into which it dissipates at the end of the metachronal wave (Fig. 7). This is consistent with the vortex-weakening mechanism found in ctenophores that harnesses fluid–structure interactions among closely spaced appendages to reduce drag (Lionetti et al., 2023).

Overall, coalescence results in higher net thrust than that produced by the same but independent pleopods (the latter yielding no inter-appendage flow interactions). Multiple coalescing pleopods overlapping their profiles during the recovery phase mitigate the impact pleopod recovery has on net thrust by inducing less drag. During the PS phase, enhanced thrust can be attributed to constructive inter-pleopod flow interactions. We found evidence of tip vortex interactions with downstream pleopods (Fig. 8B), whereby the tip vortex shed by a leading pleopod interacted with the tip of the trailing pleopod as the latter initiated its PS. Ford and Santhanakrishnan (2021b) and Garayev and Murphy (2021) associated this mechanism with enhanced thrust.

Note that because the robot was tethered, the results potentially slightly overestimate the forces, particularly thrust at the appendage and whole-body levels, due to tethering effects. Tethering causes fluid to stagnate posteriorly at the end of the RS, partially enhancing the reactive forces of the flow in the thrust direction (swimming direction) (Oliveira Santos et al., 2023). Constructive interactions of the rearward wake with downstream appendages, such as tip vortex recapture (Ford and Santhanakrishnan, 2021b; Garayev and Murphy, 2021), may be partially affected by the zero-speed condition induced by tethering. A free-swimming system would experience a higher relative velocity between the body and the wake due to their opposing motions, likely contributing to synchronizing the wake with the downstream appendages. Thus, tethering can potentially induce a temporal and spatial mismatch between the vortex-shedding patterns and the motion of the downstream appendages. Still, we found appendage Strouhal numbers around the expected biological value of *St*≈0.4, indicating similar fluid dynamics to free-swimming animals.

Overlapping the profile of several appendages via coalescence can reduce the cumulative effective surface area of multiple appendages arranged in series. But does coalescence turn the drag of three otherwise separate pleopods to that of only one? We found that during coalescence, only net thrust is produced (Fig. 9) but this is partly because the inter-pleopod phase intrinsically synchronizes the forces produced by the pleopods. However, coalescence cannot exist without the appropriate phase, making the independent contribution of pleopod grouping (i.e. overlapping profiles) and the three phases of coalescence to lower the drag indistinguishable. Future experiments designed to measure the instantaneous force output by each leg within a group could help clarify how coalescence affects their performance relative to that of their independent counterparts. Still, based on the observed flow, we can infer several possible mechanisms that would not only enhance coalescence but also potentially lower the leg-specific power required to recover.

The negative pressure regions forming on the anterior surface of pleopods during the PS (see Colin et al., 2020) could assist the later portion of the PS of an anterior pleopod by inducing a pulling force, thus taking advantage of the energy stored in the fluid to facilitate coalescence. Similarly, it is also likely that the suction forces generated behind a leading pleopod during its RS may aid in pulling the confined and trailing pleopods in a coalescing group during their RS. Recent CFD work found that grouping multiple pleopods facilitates their recovery by allowing their edge vortices to merge, causing a significant reduction in pressure in front of the appendage by redirecting the anterior flow in the stroke direction more effectively (Lou et al., 2025). This effect could reduce the energy required for the posterior pleopods to recover, potentially lowering the leg-specific cost of transport compared with that of non-coalescing pleopods. This is particularly relevant because pleopods have low inertia due to their small mass relative to the water they displace, so the additional power cost on the leading, suction-generating pleopod would be small. This marginal cost is likely outweighed by the drag-reducing benefits of coalescing several legs to minimize the effective surface area exposed to the flow.

### Pleopod bending and coalescence are complementary

Pleopod coalescence is intrinsically linked with the *B*/*L* ratio, the inter-pleopod phase and beat amplitude. Coalescence occurs by virtue of the spatial and temporal synchronization of several appendages when two or more adjacent pleopods converge and cluster together, making it possible even for stiff pleopods to coalesce. However, flexibility complements coalescence by enhancing structure–structure and fluid–structure interactions. Beyond the mechanical and hydrodynamic advantages of bending, flexible appendages are less constrained by the limited space between neighboring appendages and hard physical contact compared with their stiff counterparts (Brennen, 1974). This gain in the plasticity of the motions of the pleopods means that physical interactions are not detrimental and might instead be instrumental in promoting constructive physical interactions, such as when the legs coalesce.

While stiff rami can still coalesce, they achieve only partial lengthwise contact, often resulting in strong physical interactions that disrupt the other pleopods during the RS. These undesired effects induce a turbulent wake and cause significant disturbances in the instantaneous forces produced during coalescence (Fig. 8; Fig. S6C). In contrast, lengthwise bending allows several pleopods to overlap, conforming to the shape of adjacent pleopods to form a tight seal within a coalescing group. This configuration minimizes inter-pleopod flow (Figs 7 and 8; Fig. S5), thus reducing the impact of the RS on the overall wake to promote more consistent thrust production (Fig. S6A).

One potential challenge posed by the close contact of adjacent pleopods is the need to overcome capillary adhesion when separating the trailing pleopod from a coalescing group. Because of surface tension, a thin layer of water is confined between two adjacent legs, acting at the interface between the fluid and the wetted surface. At intermediate *Re*, the capillary effects can be significant (Butler and Vella, 2020; Lambert, 2013), potentially rendering coalescence less effective at the moment of pleopod separation. Having flexible pleopods can mitigate this problem. The rami of trailing and confined pleopods in a coalescing group (see Fig. 7 for reference) slide against each other, with the rami of the trailing leg unfurling progressively during the later phase of the RS (Fig. 7; Fig. S5). Instantaneous local lengthwise curvature measurements captured unfurling in the form of a traveling wave from the proximal to the distal section of the rami (Fig. 4A). Unfurling the trailing pleopod localizes the capillary force at the point of separation rather than the entire surface (Butler and Vella, 2020; Lambert, 2013), thus reducing the force required to separate from the coalescing group. Pleopod bending leverages coalescence from a simple byproduct of inter-pleopod phase, separation and amplitude to a mechanism optimized for minimizing disruptive hydrodynamic effects, thereby improving overall swimming performance and likely enhancing the COT.

While *P. vulgaris* displays overlapping pleopods during coalescence, appendage contact is not always present in other organisms such as ctenophores (Colin et al., 2020; Herrera-Amaya and Byron, 2023) and ciliates (Blake, 2001; Blake and Sleigh, 1974; Sleigh, 1989) whose appendage *Re* is substantially lower (*Re*<100). Still, it is interesting that all the metachronal swimmers studied to date display some form of flexible appendage that can bend during their RS, leading to the formation of an array of somewhat overlapping appendages. At higher *Re*, this configuration combines the reduced total effective surface area of individual appendages to the flow due to bending and the shielding effect from the anterior, leading appendage to reduce drag (Alben et al., 2002; Barsu et al., 2016; Gosselin et al., 2010). By enabling close physical contact between the pleopods, shrimp mitigate parasitic flow features that would develop within a loose pleopod cluster. At low *Re* where viscous forces become more important, the need to prevent inter-appendage flow is less critical. However, achieving appendage overlap during their RS by maintaining adequate appendage spacing, phase and amplitude may serve in reducing surface drag (i.e. friction) to improve the thrust-to-power ratio (Lionetti et al., 2023) and decrease the COT.

### Conclusions

Metachronal swimming is commonly found across many taxa and a wide range of scales, suggesting common biophysical mechanisms driving its success. The performance of metachronal propulsion is generally perceived from the need to maximize thrust production. However, we found that asymmetrically flexible appendages and coalescence also enhance the overall swimming performance by significantly reducing drag. Using biological and robotic experimental methods, we present several underlying morphological, functional and physical mechanisms to quantify the drag-reduction capabilities of pleopod flexibility and coalescence. We found that the curved cross-sectional profile of the pleopods enabled passive asymmetrical bending during the RS to reduce the coefficient of drag of each pleopod by 41–75.8% relative to that of stiff pleopods. During metachronal swimming, up to three pleopod pairs interact and coalesce to reduce the overall drag of the group and increase net thrust by 30.2%. Our approach successfully captured the combined contributions of all pleopods at the system level during coalescence, providing valuable insights into the biological and physical components of metachronal propulsion that will aid the development of novel bio-inspired underwater metachronal vehicles. Future work will focus on untethered swimming and appendage torque estimates to resolve the forces exerted by individual pleopods within a coalescing group during the transition from the leading to confined and trailing positions. These studies will help quantify the energetic benefits of pleopod bending and coalescence, thereby further clarifying their role in achieving high swimming performance and efficiency.

## Supplementary Material

10.1242/jexbio.249330_sup1 Supplementary information

## References

[JEB249330C1] Alba-Tercedor, J. and Alba-Alejandre, I. (2019). Comparing micro-CT results of insects with classical anatomical studies: the European honey bee (*Apis mellifera* Linnaeus, 1758) as a benchmark (Insecta: Hymenoptera, Apidae). *Microscopy*. https://analyticalscience.wiley.com/content/news-do/comparing-micro-ct-results-insects-classical-anatomical-studies-european-honey-bee-apis

[JEB249330C2] Alben, S., Shelley, M. and Zhang, J. (2002). Drag reduction through self-similar bending of a flexible body. *Nature* 420, 479-481. 10.1038/nature01232 12466836

[JEB249330C3] Alben, S., Spears, K., Garth, S., Murphy, D. and Yen, J. (2010). Coordination of multiple appendages in drag-based swimming. *J. R. Soc. Interface* 7, 1545-1557. 10.1098/rsif.2010.0171 20413558 PMC2988259

[JEB249330C4] Barsu, S., Doppler, D., Jerome, J. J. S., Rivière, N. and Lance, M. (2016). Drag measurements in laterally confined 2D canopies: reconfiguration and sheltering effect. *Phys. Fluids* 28, 107101. 10.1063/1.4962309

[JEB249330C5] Bhattacharya, R., Saha, S., Kostina, O., Muravnik, L. and Mitra, A. (2020). Replacing critical point drying with a low-cost chemical drying provides comparable surface image quality of glandular trichomes from leaves of *Millingtonia hortensis* L. f. in scanning electron micrograph. *Appl. Microsc.* 50, 15. 10.1186/s42649-020-00035-6 33580468 PMC7818294

[JEB249330C6] Blake, J. (2001). Fluid mechanics of ciliary propulsion. In *Computational Modeling in Biological Fluid Dynamics* (ed. L. J. Fauci and S. Gueron), pp. 1-51. New York, NY: Springer.

[JEB249330C7] Blake, J. R. and Sleigh, M. A. (1974). Mechanics of ciliary locomotion. *Biol. Rev.* 49, 85-125. 10.1111/j.1469-185X.1974.tb01299.x 4206625

[JEB249330C8] Brennen, C. (1974). An oscillating-boundary-layer theory for ciliary propulsion. *J. Fluid Mech.* 65, 799-824. 10.1017/S0022112074001662

[JEB249330C9] Butler, M. D. and Vella, D. (2020). Detachment in capillary adhesion: the relative roles of tilting and separation. *IMA J. Appl. Math.* 85, 673-702. 10.1093/imamat/hxaa026

[JEB249330C10] Byron, M. L., Murphy, D. W., Katija, K., Hoover, A. P., Daniels, J., Garayev, K., Takagi, D., Kanso, E., Gemmell, B. J., Ruszczyk, M. et al. (2021). Metachronal motion across scales: current challenges and future directions. *Integr. Comp. Biol.* 61, 1674-1688. 10.1093/icb/icab105 34048537

[JEB249330C11] Chen, G., Xu, Y., Yang, C., Yang, X., Hu, H., Chai, X. and Wang, D. (2023). Design and control of a novel bionic mantis shrimp robot. *IEEE/ASME Trans. Mechatronics* 28, 3376-3385. 10.1109/TMECH.2023.3266778

[JEB249330C12] Colin, S. P., Costello, J. H., Hansson, L. J., Titelman, J. and Dabiri, J. O. (2010). Stealth predation and the predatory success of the invasive ctenophore *Mnemiopsis leidyi*. *Proc. Natl. Acad. Sci. USA* 107, 17223-17227. 10.1073/pnas.1003170107 20855619 PMC2951412

[JEB249330C13] Colin, S. P., Costello, J. H., Sutherland, K. R., Gemmell, B. J., Dabiri, J. O. and Du Clos, K. T. (2020). The role of suction thrust in the metachronal paddles of swimming invertebrates. *Sci. Rep.* 10, 17790. 10.1038/s41598-020-74745-y 33082456 PMC7576154

[JEB249330C53] Combes, S. A. and Daniel, T. L. (2003). Flexural stiffness in insect wings II. Spatial distribution and dynamic wing bending. *J. Exp. Biol.* 206, 2989-2997. 10.1242/jeb.00524 12878667

[JEB249330C14] Dutcher, S. K. (2019). Asymmetries in the cilia of *Chlamydomonas*. *Philos. Trans. R. Soc. B Biol. Sci.* 375, 20190153. 10.1098/rstb.2019.0153 PMC7017335 31884924

[JEB249330C15] Fabritius, H.-O., Ziegler, A., Friák, M., Nikolov, S., Huber, J., Seidl, B. H. M., Ruangchai, S., Alagboso, F. I., Karsten, S., Lu, J. et al. (2016). Functional adaptation of crustacean exoskeletal elements through structural and compositional diversity: a combined experimental and theoretical study. *Bioinspir. Biomim.* 11, 055006. 10.1088/1748-3190/11/5/055006 27609556

[JEB249330C16] Ford, M. P. and Santhanakrishnan, A. (2021a). On the role of phase lag in multi-appendage metachronal swimming of euphausiids. *Bioinspir. Biomim.* 16, 066007. 10.1088/1748-3190/abc930 33171451

[JEB249330C17] Ford, M. P. and Santhanakrishnan, A. (2021b). Closer appendage spacing augments metachronal swimming speed by promoting tip vortex interactions. *Integr. Comp. Biol.* 61, 1608-1618. 10.1093/icb/icab112 34050744

[JEB249330C18] Ford, M. P. and Santhanakrishnan, A. (2025). Metachronal rowing provides robust propulsive performance across four orders of magnitude variation in Reynolds number. *J. R. Soc. Interface* 22, 20240822. 10.1098/rsif.2024.0822 40460863 PMC12133342

[JEB249330C19] Ford, M. P., Lai, H. K., Samaee, M. and Santhanakrishnan, A. (2019). Hydrodynamics of metachronal paddling: effects of varying Reynolds number and phase lag. *R. Soc. Open Sci.* 6, 191387. 10.1098/rsos.191387 31824735 PMC6837200

[JEB249330C20] Forward, R. B. (1988). Diel vertical migration: Zooplankton photobiology and behaviour. *Oceanogr. Mar. Biol. Annu. Rev.* 26, 361-393.

[JEB249330C21] Garayev, K. and Murphy, D. W. (2021). Metachronal swimming of mantis shrimp: kinematics and interpleopod vortex interactions. *Integr. Comp. Biol.* 61, 1631-1643. 10.1093/icb/icab052 33997904

[JEB249330C22] Garayev, K. and Murphy, D. W. (2024). Hydrodynamic scaling of metachronal swimming. *Phys. Rev. Fluids* 9, L111101. 10.1103/PhysRevFluids.9.L111101

[JEB249330C23] Gosselin, F., de Langre, E. and Machado-Almeida, B. A. (2010). Drag reduction of flexible plates by reconfiguration. *J. Fluid Mech.* 650, 319-341. 10.1017/S0022112009993673

[JEB249330C24] Guzmán, J. E. V., Hernández-Badillo, C. and Zenit, R. (2016). Experimental study of the deflections of curved plates exposed to pulsating cross-flows. *Acta Mech.* 227, 3621-3637. 10.1007/s00707-016-1687-1

[JEB249330C25] Hedrick, T. L. (2008). Software techniques for two- and three-dimensional kinematic measurements of biological and biomimetic systems. *Bioinspir. Biomim.* 3, 034001. 10.1088/1748-3182/3/3/034001 18591738

[JEB249330C26] Herrera-Amaya, A. and Byron, M. L. (2023). Omnidirectional propulsion in a metachronal swimmer. *PLoS. Comput. Biol.* 19, e1010891. 10.1371/journal.pcbi.1010891 37976322 PMC10697607

[JEB249330C27] Herrera-Amaya, A. and Byron, M. L. (2024). Propulsive efficiency of spatiotemporally asymmetric oscillating appendages at intermediate Reynolds numbers. *Bioinspir. Biomim.* 19, 066004. 10.1088/1748-3190/ad7abf 39270724

[JEB249330C28] Herrera-Amaya, A., Seber, E. K., Murphy, D. W., Patry, W. L., Knowles, T. S., Bubel, M. K. M., Maas, A. E. and Byron, M. L. (2021). Spatiotemporal asymmetry in metachronal rowing at intermediate Reynolds numbers. *Integr. Comp. Biol.* 61, 1579-1593. 10.1093/icb/icab179 34410363

[JEB249330C29] Hessler, R. R. (1985). Swimming in Crustacea. *Earth Env. Sci. T. R. So.* 76, 115-122. 10.1017/S0263593300010385

[JEB249330C30] Johnson, M. L. and Tarling, G. A. (2008). Influence of individual state on swimming capacity and behaviour of Antarctic krill *Euphausia superba*. *Mar. Ecol. Prog. Ser.* 366, 99-110. 10.3354/meps07533

[JEB249330C31] Khaderi, S. N., Den Toonder, J. M. J. and Onck, P. R. (2012). Fluid flow due to collective non-reciprocal motion of symmetrically-beating artificial cilia. *Biomicrofluidics* 6, 014106. 10.1063/1.3676068 PMC3365344 22662092

[JEB249330C32] Kils, U. (1981). *The Swimming Behavior, Swimming Performance and Energy Balance of Antarctic Krill, Euphausia superba.* Volume 3 of BIOMASS scientific series: Biological Investigations of Marine Antarctic Systems and Stocks (ed. S. Z. El-Sayed). Cambridge, UK: Scott Polar Research Institute.

[JEB249330C33] Koehl, M. A. R. (1984). How do benthic organisms withstand moving water? *Am. Zool.* 24, 57-70. 10.1093/icb/24.1.57

[JEB249330C34] Lambert, P. (ed.). (2013). *Surface Tension in Microsystems: Engineering Below the Capillary Length*. Berlin,: Springer.

[JEB249330C35] Lionetti, S., Lou, Z., Herrera-Amaya, A., Byron, M. L. and Li, C. (2023). A new propulsion enhancement mechanism in metachronal rowing at intermediate Reynolds numbers. *J. Fluid Mech.* 974, A45. 10.1017/jfm.2023.739

[JEB249330C36] Lou, Z., Herrera-Amaya, A., Byron, M. L. and Li, C. (2022). Hydrodynamics of metachronal motion: Effects of spatial asymmetry on the flow interaction between adjacent appendages. In Proceedings of the ASME 2022 Fluids Engineering Division Summer Meeting, Toronto, ON, Canada.

[JEB249330C37] Lou, Z., Tack, N., Wilhelmus, M. and Li, C. (2024). Hydrodynamics of shrimp swimming: spread-out morphing of pleopods in power stroke. In *IMECE2024*, Vol. 8. Fluids Engineering.

[JEB249330C38] Lou, Z., Tack, N., Wilhelmus, M. M. and Li, C. (2025). Edge vortex interaction minimizes drag in shrimp swimming. *Phys. Rev. Fluids* 10, 043103. 10.1103/PhysRevFluids.10.043103

[JEB249330C39] Mauchline, J. (1998). Behaviour. In *The Biology of Calanoid Copepods* (ed. J. H. S. Blaxter, A. J. Southward and P. A. Tyler), pp. 400-455. London: Academic Press.

[JEB249330C40] Murphy, D. W., Webster, D. R., Kawaguchi, S., King, R. and Yen, J. (2011). Metachronal swimming in Antarctic krill: gait kinematics and system design. *Mar. Biol.* 158, 2541-2554. 10.1007/s00227-011-1755-y

[JEB249330C41] Murphy, D. W., Webster, D. R. and Yen, J. (2013). The hydrodynamics of hovering in Antarctic krill. *Limnol. Oceanogr.* 3, 240-255. 10.1215/21573689-2401713

[JEB249330C42] Oliveira Santos, S. (2024). Shrimp as a model organism for bio-inspired underwater vehicles. *PhD thesis*, Brown University.

[JEB249330C43] Oliveira Santos, S., Tack, N., Su, Y., Cuenca-Jiménez, F., Morales-Lopez, O., Gomez-Valdez, P. A. and Wilhelmus, M. M. (2023). Pleobot: a modular robotic solution for metachronal swimming. *Sci. Rep.* 13, 9574. 10.1038/s41598-023-36185-2 37311777 PMC10264458

[JEB249330C44] Peterman, D. J. and Byron, M. L. (2024). Encoding spatiotemporal asymmetry in artificial cilia with a ctenophore-inspired soft-robotic platform. *Bioinspir. Biomim.* 19, 066002. 10.1088/1748-3190/ad791c 39255824

[JEB249330C45] Raabe, D., Sachs, C. and Romano, P. (2005). The crustacean exoskeleton as an example of a structurally and mechanically graded biological nanocomposite material. *Acta Mater.* 53, 4281-4292. 10.1016/j.actamat.2005.05.027

[JEB249330C46] Ruszczyk, M., Webster, D. R. and Yen, J. (2022). Trends in stroke kinematics, Reynolds number, and swimming mode in shrimp-like organisms. *Integr. Comp. Biol.* 62, icac067. 10.1093/icb/icac067 35662323

[JEB249330C47] Sensenig, A. T., Kiger, K. T. and Shultz, J. W. (2010). Hydrodynamic pumping by serial gill arrays in the mayfly nymph *Centroptilum triangulifer*. *J. Exp. Biol.* 213, 3319-3331. 10.1242/jeb.039271 20833925

[JEB249330C48] Sleigh, M. A. (1989). Ciliary propulsion in protozoa. *Sci. Prog.* 73, 317-331.

[JEB249330C49] Tamm, S. L. (2014). Cilia and the life of ctenophores. *Invertebr. Biol.* 133, 1-46. 10.1111/ivb.12042

[JEB249330C50] Vogel, S. (2020). *Life in Moving Fluids: The Physical Biology of Flow-Revised and Expanded Second Edition*. Princeton, NJ, USA: Princeton University Press.

[JEB249330C51] Wainwright, S. A. and Koehl, M. A. R. (1976). The nature of flow and the reaction of benthic cnidaria to it. In *Coelenterate Ecology and Behavior* (ed. G. O. Mackie), pp. 5-21. Boston, MA: Springer US.

[JEB249330C52] Wei, A., Guo, Z. and Guo, F. (2022). Unveiling the mechanism behind the asymmetric bending compliance of thin-walled U-shaped strips: a study inspired by plant leaves. *Acta Mech. Solida Sin.* 36, 156-165. 10.1007/s10338-022-00361-0

